# Developing a 3D Model Culture of an EBV+/CD30+ B-Anaplastic Large Cell Lymphoma Cell Line to Assay Brentuximab Vedotin Treatment

**DOI:** 10.3390/antib14040098

**Published:** 2025-11-10

**Authors:** Paolo Giannoni, Gabriella Pietra, Orlando Izzo, Giuseppina Fugazza, Roberto Benelli, Alessandro Poggi, Mauro Krampera, Chiara Utzeri, Monica Marchese, Marco Musso, Paola Visconti, Daniela de Totero

**Affiliations:** 1 Department Experimental Medicine, University of Genoa, 16132 Genoa, Italy; paolo.giannoni@unige.it (P.G.); gabriella.pietra@unige.it (G.P.); 2 Pathology and Experimental Immunology Unit, IRCCS Ospedale Policlinico San Martino, 16132 Genoa, Italy; orliolli93@gmail.com; 3 Clinic of Hematology, Department of Internal Medicine, University of Genoa, 16132 Genoa, Italy; giuseppina.fugazza@unige.it; 4 Hematology and Oncology Unit, IRCCS Ospedale Policlinico San Martino, 16132 Genoa, Italy; 5 Molecular Oncology and Angiogenesis Unit, IRCCS Ospedale Policlinico San Martino, 16132 Genoa, Italyalessandropoggi1952@gmail.com (A.P.); 6 Hematology and Bone Marrow Transplant Unit, Section of Biomedicine Innovation, Department of Engineering for Innovative Medicine (DIMI), University of Verona, 37100 Verona, Italychiara.utzeri@univr.it (C.U.); 7 Biological Resources Center, IRCCS Ospedale Policlinico San Martino, 16132 Genoa, Italy; monica.marchese@hsanmartino.it (M.M.); marco.musso@hsanmartino.it (M.M.); paola.visconti@hsanmartino.it (P.V.); 8 Molecular Pathology Unit, IRCCS Ospedale Policlinico San Martino, 16132 Genoa, Italy

**Keywords:** antibody–drug conjugates, CD30 expression, brentuximab vedotin, B-anaplastic large cell lymphoma, 3D culture models

## Abstract

Background/Objectives: Three-dimensional (3D) in vitro cell culture models have recently stimulated great interest since they may have more pre-clinical value than conventional in vitro 2D models. In fact, 3D culture models may mimic the in vivo biophysical 3D structure of tumors and cell-to-cell interaction, thereby representing a more useful approach to testing drug responses. In this study we have developed a 3D culture model of an EBV+/CD30+cell line, D430B, previously characterized as an Anaplastic Large Cell Lymphoma of B phenotype (B-ALCL), to determine the cytotoxic activity of the antibody–drug conjugate Brentuximab Vedotin. Methods: By using of ultra-low attachment plates, we developed D430B spheroids that appeared particularly homogenous in terms of growth and size. Results: Brentuximab Vedotin treatment (1 to 20 μg/mL) turned out to be significantly cytotoxic to these cells, while the addition of the anti-CD20 chimeric antibody Rituximab (10 μg/mL) appeared almost ineffective, even though these cells express CD20. Moreover, when we co-cultured D430B cells with stromal cells (HS5), to re-create a microenvironment representative of neoplastic cell/mesenchymal cell interactions within the lymph node, we observed a significant, although faint, protective effect. Conclusions: This simple and reproducible method of generating D430B-ALCL spheroids to evaluate their response to Brentuximab Vedotin treatment, as here described, may provide a valuable preliminary tool for the future pre-clinical screening of patients’ primary lymphoma cells or the development of novel therapies for this type of pathology and related diseases.

## 1. Introduction

Brentuximab Vedotin (BV) is an antibody–drug conjugate (ADC) targeting the CD30 antigen. Brentuximab Vedotin is composed of the humanized IgG1 mAb SGN-35, anti-CD30, combined with a synthetic anti-mitotic agent, monomethyl auristatin E (MMAE), that potently inhibits tubulin polymerization causing apoptotic cell death [1,2,3]. CD30 is a member of the TNFR family and was originally identified as a cell-surface marker of Reed–Stemberg and Hodgkin cells of classical Hodgkin lymphomas (cHL) [4,5,6]. CD30, however, is also expressed at variable degrees in various types of lymphomas, such as peripheral T-cell lymphomas (PTCLs), some cutaneous T-cell lymphomas (CTCLs), and B-cell lymphomas such as diffuse large B-cell lymphomas (DLBCLs). Since CD30 is barely detectable in healthy tissues, it represents an ideal target for immuno-based therapy [7,8,9]. Over the last 20 years, several therapeutic tools have been developed to target the CD30 antigen, such as monoclonal antibodies, immunotoxins, and radio-labeled antibodies; however, these approaches have demonstrated limited success in clinical phase I studies [10,11,12,13,14,15]. In the last few years, the results of many pre-clinical in vitro and in vivo studies have prompted the fast approval of using BV to treat lymphoma patients with relapsed/refractory (R/R) Anaplastic Large Cell Lymphoma (ALCL) and cHL [16,17]. Brentuximab Vedotin has led to revolutionary changes in the treatment of lymphomas, especially in NHL, with consistent efficacy and safety, during first-line and salvage treatment [15,18]. In this study we aimed to develop a 3D culture model to test the efficacy of BV in an EBV^+^/CD30^+^ Anaplastic Large Cell Lymphoma (ALCL) cell line of the B phenotype (D430B), which we established previously [19]. Although the application and efficacy of BV in T-cell type ALCL and in cHL have been largely exploited and clinically validated over the course of the last decade, the FDA only recently approved BV as a treatment for R/R DLBCL in combination therapy (with lenalidomide and/or Rituximab), based on the ongoing ECHELON 3 trial (ClinicalTrials.gov.identifier:NCT04404283:February 2025). Indeed, today there is a wide consensus that lymphomas with an anaplastic morphology, characterized by a B cell phenotype and ALK negativity, represent a subtype of Diffuse Large B-cell Lymphoma (DLBCL) [20,21]. Since the D430B cell line belongs to this latter subtype and it has been previously used in pre-clinical conventional culture with CD30-immunotoxins, and in vivo in an SCID-mouse model [10,19], we believe that moving from cell monolayer cultures to 3D cultures could increase the translatability of pre-clinical experiments. The exploitation of new-generation ADCs in this setting could further contribute to the development of novel therapeutic strategies. In our case, D430B spheroids turned out to be highly sensitive to the cytotoxic activity of BV. Keeping in line with the interest of applying BV treatment in combination with additional drugs [22,23,24], we further evaluated the simultaneous administration of BV with Rituximab (Rixathon: a humanized chimeric anti-CD20 moAb) in D430B cells. Rituximab, however, appeared almost ineffective, and the contemporary use of BV with Rituximab did not evidence an increase in the BV cytotoxic effect.

## 2. Materials and Methods

### 2.1. Cell Lines Culture

The D430B cell line was established as previously reported [19]. The D430B cell line is a human monoclonal EBV-infected cell line capable of growing in SCID mice developing solid tumors with the morphology of CD30+ ALCL. D430B was cultured in suspension in vitro in complete medium (RPMI 1640 supplemented with 10% heat-inactivated fetal calf serum (FCS); 1% penicillin-streptomycin, and 2% L-Glutamine) (Lonza, Walkersville, MD, USA)) in 25 or 75 cm^2^ flasks. The HS5 cell line (ATCC^®^ CRL-11882^TM^) is a human stromal cell line [25] often used as a model for the haemato-lymphopoietic microenvironment. HS5 was expanded in a complete medium in adherence in flasks. When the cells reached 80% of confluence, they were detached using 1X trypsin-EDTA (Euroclone S.p.A., Pero, Milan, Italy), divided, frozen, and utilized in the experiments. Both D430B and HS5 tested negative for mycoplasma. The D430B cell line has been deposited at the ICLC; IRCCS Ospedale Policlinico San Martino, Genoa, Italy.

### 2.2. Short Tandem Repeat Analysis

Short tandem repeat (STR) profiling was performed to verify the authenticity of the D430B cell line, using the services provided by Banca Cellule Interlab Cell Line Collection (ICLC; IRCCS Ospedale Policlinico San Martino, Genoa, Italy) as previously described [26]. The STR profile of the cell line was determined: fifteen highly polymorphic STR loci plus amelogenin (Cell IDTMSystem, Promega Italia, 20126 Milano, Italy) were used. Amplified fragments were obtained using the ABI PRISM 3100 Genetic Analyzer (Applied Biosystem, Forster City, CA, USA). Data were analyzed using Gene Mapper software, version 3.2 (Applied Biosystems, Whaltman, MA, USA).

### 2.3. Cytogenetics Analysis of D430B Cell Line

We performed a chromosome study in the D430B cell line to determine its karyotype. Briefly, cells were exposed to colcemid (0.04 µg/mL) for 30 min at 37 °C and subjected to hypotonic treatment (0.075 M KCl) for 15 min at room temperature. Cells were then fixed in a methanol and acetic acid (3:1 volume/volume) mixture for 15 min and then washed three times in a fixative. The slides were air-dried. Karyotyping was carried out on QFQ-banded chromosomes and was reported using the International System for the Human Cytogenetic Nomenclature [27,28]. Fluorescent in situ hybridization (FISH) was performed using the Vysis LSI ALK Dual Color, Break Apart Rearrangement Probe Kit (Bio-Optica S.p.A. Milan, Italy) [29] following manufacturer’s instructions.

### 2.4. Analysis of the D430B Cell Line Phenotype and of the Brentuximab Vedotin and Rituximab Binding via Flow Cytometry

The D430B cell line was analyzed via flow cytometry to determine the expression of the CD30, CD19, CD20, CD22 antigens and kappa and lambda light Ig chain. Phenotypical analysis was therefore performed by staining the cells with conjugated antibodies specific for membrane antigens such as anti-human-CD19-PE, -CD20-FITC, -CD22-PE, and anti-Ig- Kappa- or -Lambda-FITC (Immuno-Tools GmbH, Friesoythe, Germany), as previously described [30]. The expression of CD30 was then determined with the primary anti-CD30 moAb (BerH2) (a gift from Prof. B.Falini, Perugia) followed by a secondary anti-mouse IgG-PE (Immunotools) conjugated antibody, as previously described [19]. The HS5 cell line was also used as a negative control for the expression of the CD30 antigen and stained as described above. HS5 cells were also stained with anti-human CD73-FITC, CD105-PE, and CD90-APC, (Immunotools) fluorochrome conjugated moAbs. Moreover, the binding of BV or of Rituximab to D430B cells was further evaluated. Brentuximab Vedotin (Adcetris^®^) and Rituximab (Rixathon) were obtained as a leftover of the therapeutic preparation used for patients (Pharmacy Unit of IRCCS Ospedale Policlinico San Martino, Genoa, Italy). D430B or HS5 cells were incubated with BV (10 μg/mL) or Rituximab (10 μg/mL) for 30 min at 4 °C. After two washes with PBS+ 2% FCS, 50 μL of anti-human IgG FC-PE-conjugated antibody (Jackson Immune Research Laboratory Inc, Europe LTD, Cambridge, UK) was added. Following 30 min of incubation, samples were acquired using an FACS-CANTO cytofluorimeter (Beckton Dickinson; Franklin Lakes, NJ, USA) or FACS Gallios, three lasers, and 10 colors (Beckman Coulter Life Sciences, Milan, Italy). At least 10,000 events were acquired for each sample and the data were analyzed using FlowJo software version 10.

### 2.5. Evaluation of Brentuximab Vedotin Cytotoxic Activity in Conventional 2D Model by DioC6 and the Alamar Blue Assay

The cytotoxic activity of BV (10 μg/mL) to D430B cells (5 × 10^3^ cells/mL) cultured with or without HS5 cells (10 × 10^3^ cells/mL) in a 24 well plate for 72 h or 96 h was determined by evaluating changes in the inner mitochondrial membrane potential by staining the D430B cells at 37 °C for 15 min with 40 nM of DiOC6 (3,3 dihexylocarbocyanine iodide; Sigma, Milano Italy), a green fluorescent dye, as we previously described [30]. Cytofluorographic analysis was then performed using an FACS-CANTO cytofluorimeter (Beckton Dickinson), and corresponding data analysis was performed using FlowJo software version 10. Proliferation of D430B cells co-cultured or not with HS5 in a 24 well plate, with or without BV treatment (10 μg/mL) for 96 h, was also assessed using Alamar Blue^TM^ (Invitrogen; Milan, Italy). Briefly, supernatants containing the control (only D430B cells) or D430B cells co-cultured with HS5 were collected and centrifuged (400× *g*). Cell pellets were resuspended in 500 μL of complete medium, supplemented with 10% Alamar Blue^TM^. The cells were maintained at 37 °C for 4 h, at dark, and then re-centrifuged as described above. Aliquots of retrieved supernatants (200 μL) were spectrophotometrically assessed, evaluating absorbance at 570–600 nm, according to the manufacturer’s instruction, in a Spectra MR Dynex apparatus (DYNEX Technologies, Chantilly, VA, USA).

### 2.6. Preparation of Spheroids

Several attempts have been made to define the most suitable number of cells per spheroid. Different types of 96-well U-bottom plates were used, such as Standard low attachment (LONZA), ultra-low attachment (ULA) (Microplate; 96 well, U-Bottom Cellstar^®^, Cell-repellent surface; cod. 650970; Greiner Bio-One GmbH, Maybachstr 2, 72636 Frickenhausen, Germany) using different numbers of cells (5 × 10^3^–20 × 10^3^: 5–20K cells) in 200 µL of complete medium. Whenever used, type 1 collagen was supplemented, at the concentration of 1.0–10.0 µg/mL (PureCol^®^EZ Gel; cod. 5074-G; Advanced BioMatrix Inc.; Carlsbad, CA, USA) (Supplementary Figure S1; Panel A). We also applied the hanging-drop method, delivering 10 × 10^3^ cells/drop (50 μL of complete culture medium) [31,32,33]. For each method we allowed spheroid formation for at least 48 h prior to any additional treatment. When we further generated hybrid spheroids, we co-cultured 3 × 10^3^ D430B cells with 2 × 10^3^ HS5 cells in 200 μL of complete medium (RPMI 1640 + FCS 10%) in 96-well U bottom ULA plates.

### 2.7. Brentuximab Vedotin Cytotoxicity in a 3D Culture Model

Standard and/or hybrid spheroids were generated by seeding 5 × 10^3^ D430B cells or 3 × 10^3^ D430B cells + 2 × 10^3^ HS5 cells, respectively, in 200 µL of complete medium, in each well of a 96-well ULA plate and cultured in the incubator at 5% CO_2_ and 37 °C for 48 h. After 48 h, 50 μL of completed medium with or without BV (1; 2,5; 5; 10; 20 µg/mL) or Rituximab (10 µg/mL) or BV+Rituximab (10 μg each) was added. The final volume was therefore 250 μL/well. In some experiments we utilized the CELLCYTE X^TM^ imaging recorder (Cytena, Breisgau, Germany) to perform time lapsed imaging (0, 24, 48, and 72 h). In this case we added the C.LIVE Tox green probe (Cytena, Breisgau, Germany) to each well at the final concentration of 50 nM to evaluate apoptotic cells in terms of fluorescence mean intensity; assessment of the area of each spheroid was also simultaneously calculated using the CELLCYTE Studio software (https://www.cytena.com/new-cellcyte-x-software-january-2022/, accessed on 15 January 2022). The evaluation of cell viability in spheroids treated or untreated with BV was also determined by using the CellTiter-Glo^®^ luminescent assay in accordance with the manufacturer’s instructions (Promega Italia s.r.l.; Milan, Italy). Through this assay the presence of different concentrations of ATP, a marker of metabolically active/living/proliferating cells, was determined. Additional sets of sample images were acquired using a Nikon Digital Sight DS-5 Mc camera mounted onto an inverted Olympus CKX-41 microscope (Olympus Corp., Tokyo, Japan). The spheroid areas were determined using Image J software tools (Image J free software, version 1.48; http://imagej.net/ij, accessed on 15 January 2022). Spheroids’ images were further acquired every 24 h to assess spheroid formation and growth for up to 192 h (8 days). Moreover, the contribution of D430B cells to the area of hybrid spheroids was determined by subtracting the HS5 area from the total spheroid area, as also detailed in Supplementary Figure S2.

### 2.8. Evaluation of Apoptosis in Spheroids via AnnexinV/PI Staining

Apoptosis induced after 72 h of BV treatment (10 μg/mL) in spheroids (D430B cells alone) or in hybrid spheroids (D430B cells + HS5 cells) was also evaluated using AnnexinV/PI staining. Briefly, each spheroid, untreated or treated, was picked up from each well and gently transferred in an FACS tube. After dissociation via pipetting up and down several times, the cells were stained with FITC Annexin V and PI according to the manufacturer’s instructions (Immunostep S.L., Salamanca, Spain). Briefly, the binding buffer (BB) was diluted 1:10 in distilled water. Cells were washed twice in PBS and resuspended in BB (100 μL). Then, 5 μL of FITC Annexin V and 5 μL of PI were added to the cell suspension. The cells were incubated in the dark for 15 min at room temperature. After incubation, 400 μL of BB was added to the cells. Flow cytometry analyses were conducted within one hour of PI staining. The samples were acquired using the FACS Gallios, three lasers, and 10 colors (Beckman Coulter). Data analysis was performed using FlowJo software version 10.

### 2.9. Statistical Analysis

Whenever indicated, Student’s *t*-test was applied to evaluate the statistical significance of the different experimental conditions (*: 0.01 < *p* ≤ 0.05; **: 0.001 < *p* ≤ 0.01; ***: *p* ≤ 0.001).

## 3. Results

### 3.1. Determination of CD30 Antigen Expression Level in D430B Anaplastic Large Cell Lymphoma Cells via Flow Cytometry Analysis

**Figure 1:**
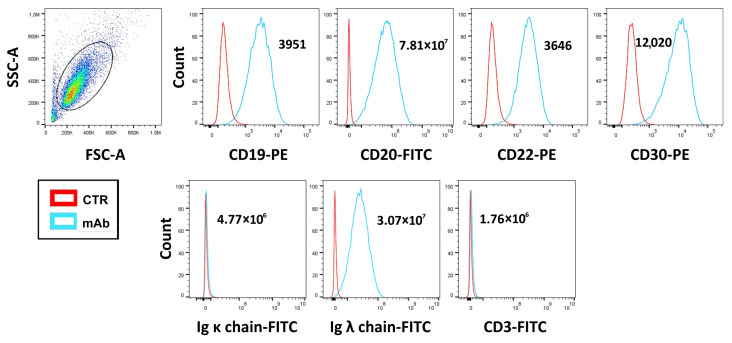
**Determination of the phenotypic profiles of the D430B cell line via cytofluorimetric analysis**. D430B cells were stained with specific antibodies targeting the indicated antigens. The first dot plot on the left indicates the physical parameters (forward scatter, FSC; and side scatter, SSC) of D430B and the gate of the analysis. The histograms show the expression of CD19, CD20, CD22, CD30, Ig kappa and Ig lambda light chains, and CD3 in D430B cells. Mean fluorescence intensity (m.f.i.) values are indicated in each plot.

Flow cytometry analysis revealed the high expression of the CD30 antigen by the D430B cell line (mean fluorescence intensity: m.f.i. = 12,020) (Figure 1). These cells also clearly express CD19, CD20, CD22, and only the IgG lambda light chain (Figure 1), thus confirming their B phenotype and monoclonality as previously demonstrated [19].

### 3.2. Authenticity of the D430B Cell Line

The authenticity of the D430B cell line was verified through short tandem repeat (STR) profiling using the services provided by the Banca Cellule Interlab Cell Line Collection (IRCCS Ospedale Policlinico San Martino, Genoa, Italy). The resulting STR profile, shown in Table 1, demonstrated the unique profile of these cells, relative to the profiles present in the reference databases.

### 3.3. Cytogenetics of the D430B Cell Line

**Figure 2:**
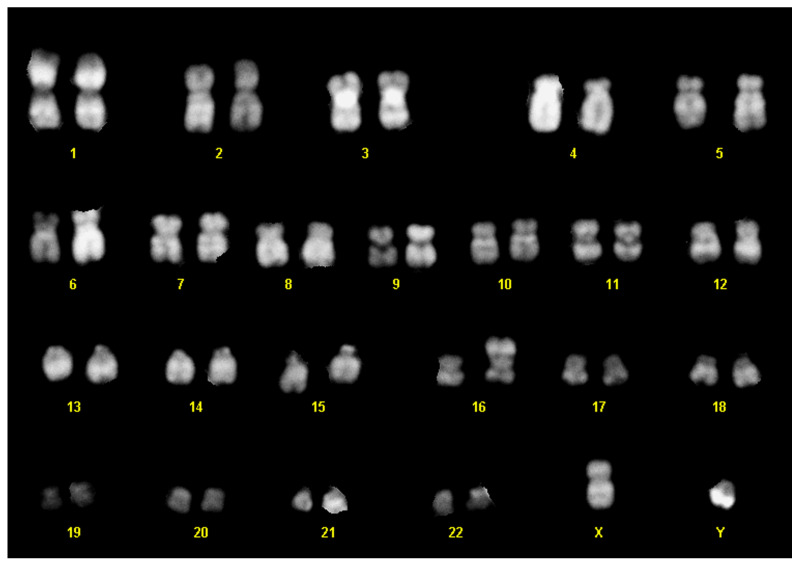
Karyotype of the D430B cell line.

Cytogenetics analysis performed in D430B cell line evidenced a karyotype substantially equivalent to the previously described one [19] with 46, XY, der (16) t (11,16) (q13; p13) (analyzed metaphases: *n* = 20), with subsequent partial trisomy of the long arm of chromosome 11 (Figure 2). We also performed fluorescent in situ hybridization to confirm that the ALK rearrangement was negative, as expected from the karyotype, which did not evidence the typical translocation found in ALK + ALCL t (2;5) (p23: q35) (unpublished material).

### 3.4. Cytofluorimetric Analysis of the Binding of Brentuximab Vedotin and Rituximab to D430B Cells

**Figure 3:**
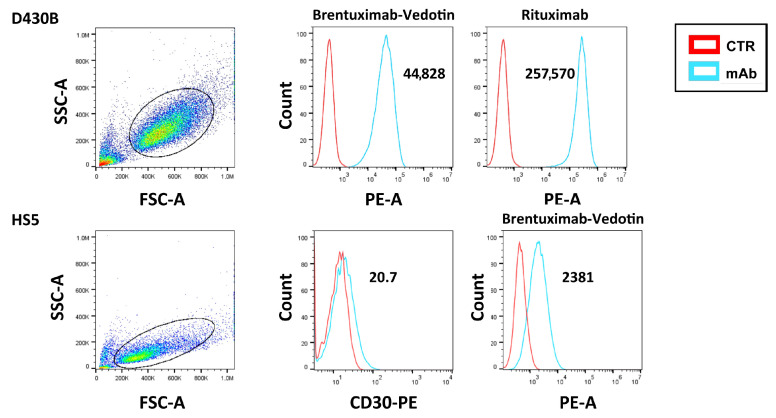
**Brentuximab Vedotin and Rituximab binding to the D430B and HS5 cell lines.** The histograms show the binding affinity of BV and Rituximab to D430B cells and to HS5 cells. Mean fluorescence intensity (m.f.i.) values are indicated in each plot.

The binding affinity of BV and Rituximab to D430B cells was confirmed via flow cytometry, as shown in Figure 3. Moreover, HS5, used as control for the D430B cells and in the co-culture experiments, showed a weak expression of CD30. HS5 instead expressed typical markers of stromal cells, such as CD73, CD105, and CD90, as shown in Supplementary Figure S3.

### 3.5. Establishment of D430B Spheroids

**Figure 4:**
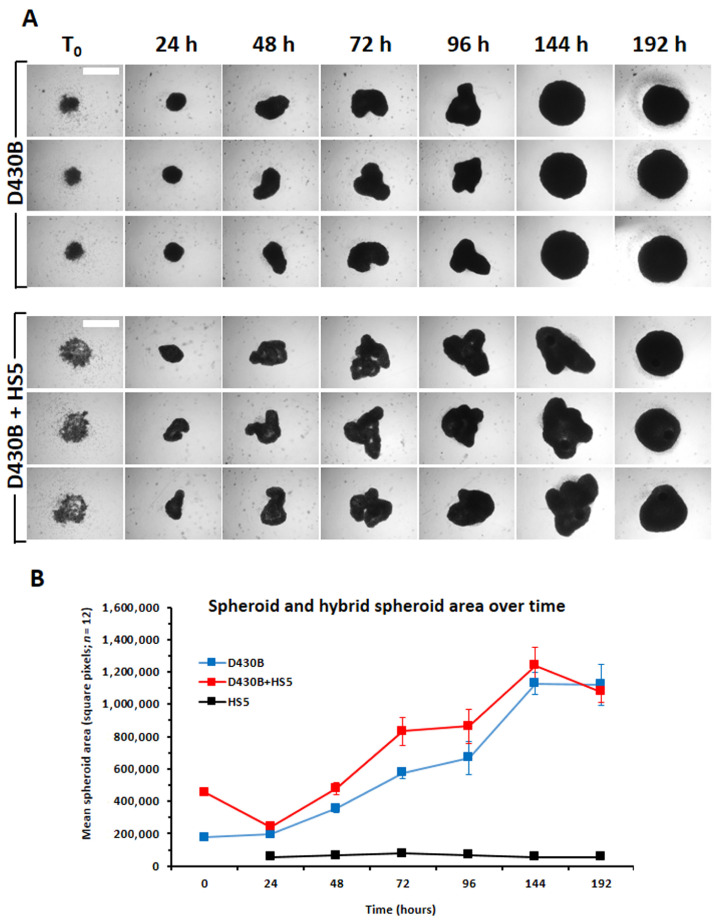
Observations of D430B spheroids and D430B+HS5 spheroids (hybrid) formation over time (0–8 days) in 96-well U-bottom ULA plates. (**A**) Seeding 5 × 10^3^ D430B (upper) or 3 × 10^3^ D430B + 2 × 10^3^ HS5 cells/well (lower) in ULA plates in 200 μL of complete medium allowed the formation of spheroids appearing quite homogeneous already at early timings (24–48 h). A compact, more defined and enlarged round shape was reached after a longer culturing period (6–8 days). The two sets of images (D430B and D430B+HS5) depicted are three representative samples of 12 spheroids (blue squares) or hybrid spheroids (red squares) followed over time. White bar: 100 µm (**B**) The depicted curves indicate the growth of the spheroid/hybrid spheroid areas over the course of experimental timings. Each time point for each type of spheroid is the mean ± SD of 12 samples. Growth was calculated by determining the number of square pixels for each spheroid area by the ImageJ software (version 1.48). The extrapolated area for the HS5 components (black squares) in hybrid spheroids (calculated as indicated in Supplementary Figure S2) revealed the conservation of a stable dimension from 24 h onward.

Several attempts have been made to define the most proper number of cells and the most suitable methods for establishing D430B spheroids, such as the hanging-drop technique or seeding the cells in several types of culture plates in the absence or presence of ECM components such as collagen. Standard low attachment U-bottom culture plates allowed the generation of regularly shaped spheroids after only 48 h when 20 × 10^3^ (20K) D430B cells/per well were cultured in 200 µL of complete medium; supplementation of 1.0–10.0 µg/mL of type 1 collagen did not ameliorate either the shaping or the timings of spheroid generation (Supplementary Figure S1, Panel A). Via the hanging-drop method [31,32,33], spheroid consistency, with the same timing, was attained using 10 × 10^3^ cells/drop. This approach, although economically advantageous, was assumed to be hardly practical for more extensive applications, mainly due to its limitations in terms of culturing volumes, in exposing samples to treatment variations, and in sample handling (Supplementary Figure S1; Panel B). We then assessed spheroid formation in ultra-low attachment (ULA) U-bottom well plates, which provided the best combination of minimal cell handling, culturing volumes requirements, and the number of cells required to obtain stable spheroids and timing (5 × 10^3^ D430B cells/well; Supplementary Figure S1; Panel C). The marked homogeneity of spheroids among the culture wells appears a key feature for consistency and repeatability of the assay to plan the in vitro assessment of a drug’s effect and consequent in vivo application in personalized medicine. This latter methodological approach was therefore chosen as the most convenient for our experimental setting and applied in the subsequent investigations. Figure 4A shows representative images of the formation over time of D430B spheroids or hybrid spheroids generated by co-culturing D430B cells and mesenchymal cells (HS5 cell line). Rather compact and homogeneous cell micro-masses were observed over the course of 24–96 h, reaching a more uniform rounded shape after 6–8 days of culture. Figure 4B provides further evidence of the substantially linear growth of the spheroids up to day 6, reaching a plateau between 6 and 8 days of culturing.

### 3.6. Brentuximab Vedotin and Rituximab Cytotoxic Effect on D430B Spheroids

**Figure 5:**
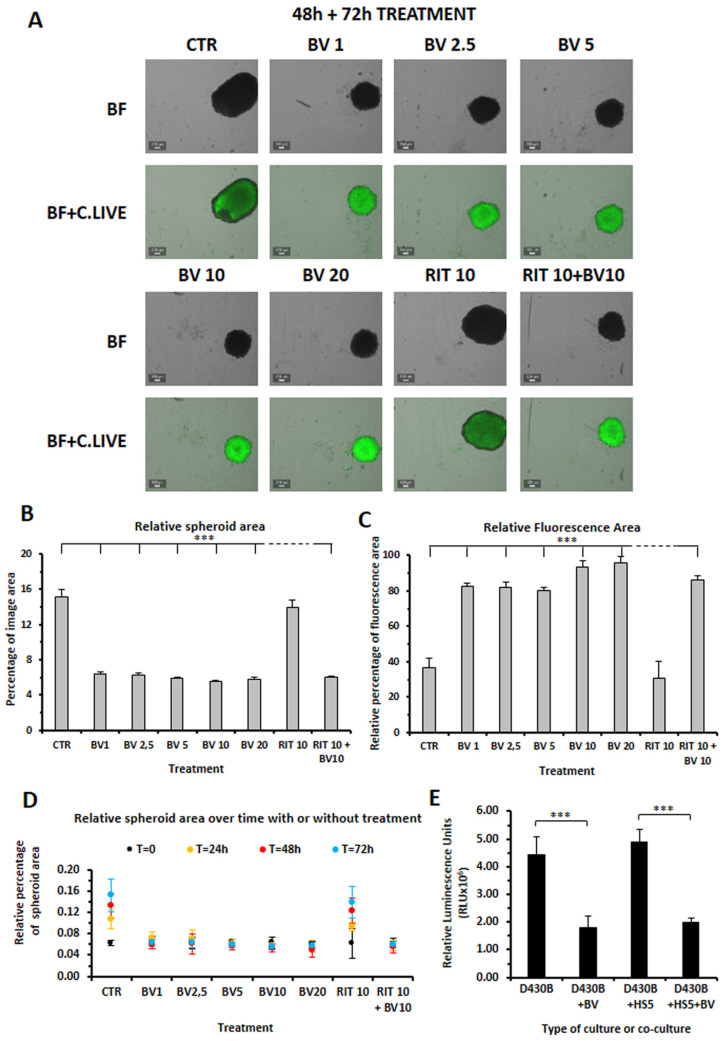
D430B spheroids untreated or treated with different doses of Brentuximab Vedotin (1–20 μg/mL) or with Rituximab (10 μg/mL) for 72 h. Cytotoxic activity was determined in terms of the reduction in spheroids area, fluorescence intensity, or cell viability. (**A**) BV treatment strongly reduced D430B spheroids formation while Rituximab was almost ineffective. Each image is representative of 4 spheroids tested for each treatment. BV treatment induced intense fluorescence as detected by the DNA-sensitive C.LIVE Tox green dye while Rituximab was almost ineffective. Each image is representative of the 4 spheroids tested for each treatment, shown here as the merge of bright-field and green fluorescence. (**B**) The histograms represent the mean ± SD of 4 spheroids whose relative spheroid areas have been calculated. (**C**) The histograms represent the mean ± SD of 4 spheroids whose relative fluorescence area, as determined through the C.LIVE Tox green dye, has been calculated. (**D**) Evaluation of the formation of D430B spheroids (relative areas) at each experimental timing. The values represent the mean ± SD of 4 spheroids determined at each time point. All images were recorded using the CELL-CYTEX^TM^ and determinations were performed by the CELLCYTE studio software. (**E**) The histograms depict the luminescence-relative units related to the ATP concentrations, which are a marker of metabolically active cells, in treated/untreated different spheroids types, as determined via the CellTiter-Glo^®^ assay. The values represent the mean ± SD of 6 spheroids of each type and treatment and *** indicates *p* ≤ 0.001.

Next we evaluated the cytotoxic effect of BV and Rituximab on D430B spheroids. As shown in Figure 5, BV was strongly cytotoxic to the D430B spheroids at the different doses used (1–20 μg/mL), while when we added Rituximab alone, we observed very low cytotoxic activity. After 72 h of BV treatment, the D430B spheroid areas appeared significantly reduced; in parallel, their fluorescence intensity, detected using the DNA-sensitive binding dye C.LIVE Tox green, was strongly enhanced. By contrast, Rituximab alone did not induce any significant variations in size or fluorescence intensity (Figure 5A–C). When we further analyzed the variations in the relative area of the spheroids over time (from time 0 to 72 h), as shown in Figure 5D, we could also appreciate that the growth of D430B spheroids was significantly inhibited in those treated with BV. The strong cytotoxic activity of BV (10 μg/mL) with respect to the D430B spheroids was also confirmed through the evaluation of the ATP content with the CellTiter-Glo^®^ assay, which revealed an around 60% loss of cell viability compared to that of the untreated spheroids (Figure 5E). The same results were also obtained when assaying the hybrid spheroids (Figure 5E).

### 3.7. Determination of Brentuximab Vedotin’s Cytotoxicity on D430B Cells Co-Cultured with Stromal HS5 Cells in 2D and in 3D Models

**Figure 6:**
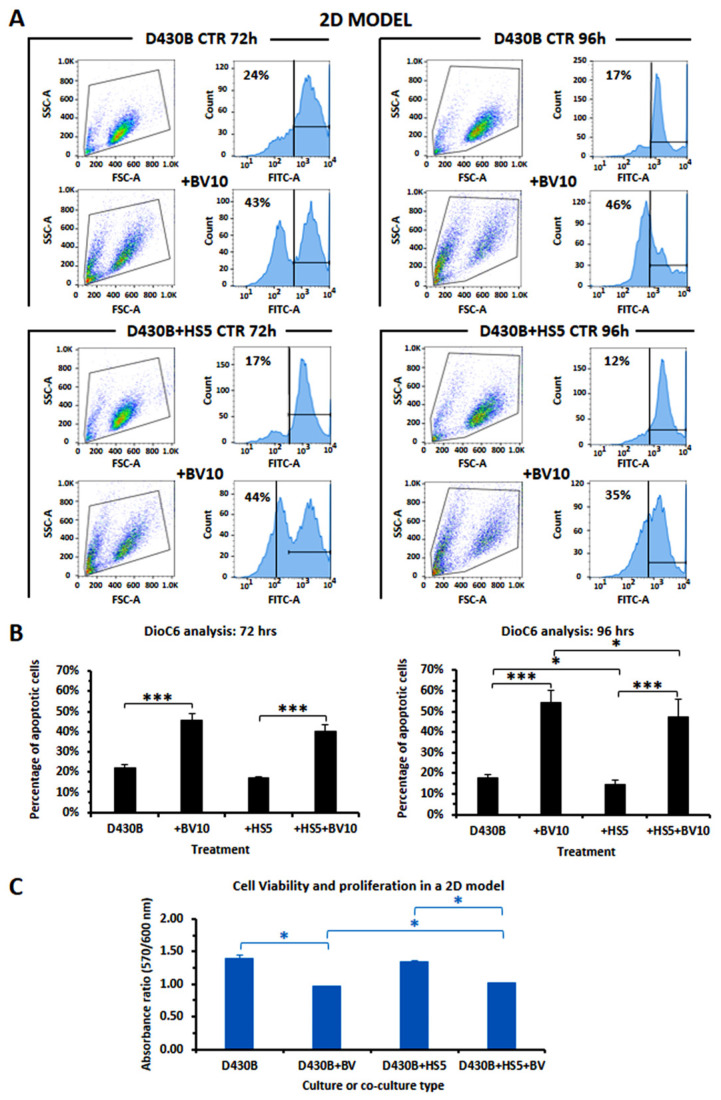
Evaluation of apoptosis and of viability of D430B cells, cultured alone or with HS5, treated or untreated with Brentuximab Vedotin, in a conventional 2D model. (**A**) The presence of HS5 in D430B cultures reduced the apoptotic activity of BV at 96 h, as detected via DioC6 staining. The histograms are representative of four independent samples. (**B**) The histograms depict the mean percentage ± SD of D430B apoptotic cells cultured with/without HS5 and treated/untreated with BV10 (10 µg/mL) for 72 or 96 h, as determined in 4 independent samples. (**C**) Viability of D430B cells cultured with/without HS5 cells as assayed by the Alamar blue test. The histograms represent the mean ± SD of one experiment performed in duplicate. Statistical significance is indicated (*): * is *p* ≤ 0.05; *** is *p* ≤ 0.001.

We further assessed if interactions between lymphoma cells and cells of the microenvironment could influence D430B cells’ response to BV when they were co-cultured with the stromal cell line HS5, in classical flat plates (2D model) as well as in the 3D model that we have here described. As shown in Figure 6A,B, the values of apoptosis, as detected via DioC6 staining in the conventional 2D culture model, demonstrated that the cytotoxicity of BV treatment toward D430B cells was weakly reduced by co-culturing the lymphoma cells with the HS5 stromal cells after 96 h of treatment (44% versus 35%). In addition, evaluation of viability via the Alamar Blue assay also confirmed that the presence of HS5 cells significantly inhibited the cytotoxic effect of BV on D430B cells (Figure 6B).

**Figure 7:**
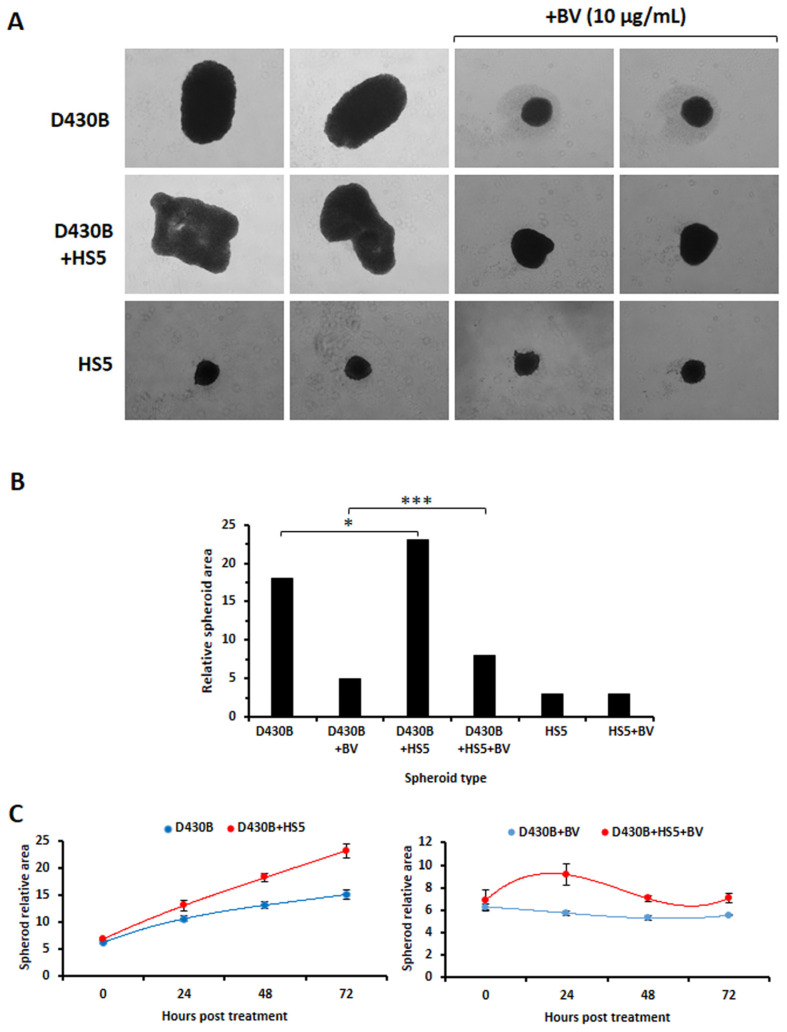
**Determination of Brentuximab Vedotin’s activity in spheroids (D430B only) and hybrid spheroids (D430B + HS5).** (**A**) BV strongly inhibits the growth of spheroids and of hybrid spheroids. Images are representative of 6 spheroids (treated or untreated) generated in each well for each type of sample (D430B, D430B+HS5, HS5). (**B**) The histograms represent the mean of 6 spheroids for each cell type treated or untreated with BV (10 μg/mL). (**C**) Comparison of spheroids and hybrid spheroids formation, with/without BV treatment (10 ug/mL), over the course of the experiment. The values represent the mean ± SD of 4 spheroids determined at each time point. * indicates *p* ≤ 0.05 and *** *p* ≤ 0.001.

As further shown in Figure 7A,B, the BV treatment, at 10 μg/mL, strongly inhibited the growth of the D430B spheroids, with a reduction of almost 70%. The presence of HS5 cells, however, appeared to enhance the relative area values of these hybrid spheroids, and a statistically relative difference was observed for the hybrid spheroids under treatment, as evaluated by subtracting the HS5 area. Apparently, these HS5 spheroids did not show a marked reduction in size in the presence of BV (Figure 7A,B). As reported in Supplementary Figure S2, which also summarizes the procedures employed to generate different types of spheroids as well as the treatment timings (A-B), a more detailed evaluation of the acquired spheroid images confirmed that the enlargement of the spheroid total area was really due to D430B cells and not to HS5 (C-D). In addition, the comparison over time determined via the CELLCYTE X^TM^ imaging recorder also highlighted a proliferative support exerted by HS5, which was, however, strongly counteracted by BV administration (Figure 7C).

**Figure 8:**
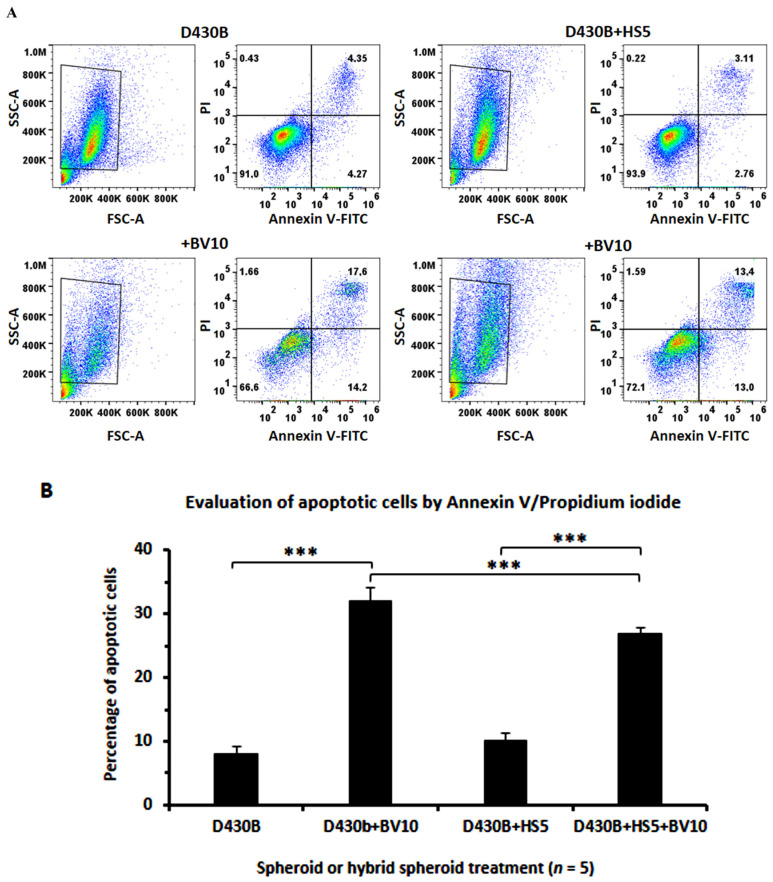
Apoptosis induced by Brentuximab Vedotin in spheroids and hybrid spheroids as evaluated via Annexin V/PI staining. (**A**) The evaluation of apoptosis, as detected via Annexin/PI staining in spheroids and hybrid spheroids, treated/untreated with BV for 72 h, revealed that HS5 cells exerted a protective effect for D430B cells under BV treatment. Dot plots are representative of 5 spheroids assessed per type/treatment. (**B**) The histograms, summarizing data on apoptosis induced by BV treatment in D430B spheroids and hybrid spheroids, confirm that the HS5 cells had a significative rescuing effect with respect to BV-induced D430B apoptosis. Bars are the mean percentage ± SD of apoptotic cells in 5 stained spheroids per type. *** indicates *p* ≤ 0.001.

As shown in Figure 8A, the evaluation of the induction of apoptosis by BV (10 μg/mL) in spheroids and in hybrid spheroids using Annexin V/PI staining also highlighted a weak protective effect induced by the presence of stromal cells (apoptotic: 26% versus 31%). Figure 8, summarizing the results of five samples per spheroids type or treatment, also indicated that HS5 exerted a significant protective effect with respect to BV-induced apoptosis.

## 4. Discussion

CD30, also called TNFRFS8, was originally considered as a marker of Hodgkin lymphoma, but its expression was later also observed in other different types of lymphoma [9,34]. We here describe the establishment of a 3D culture model of an EBV-infected B cell line (D430B), expressing the CD30 antigen and previously shown to be capable of growing in SCID mice developing solid tumors with the morphology of CD30^+^ ALCL lymphoma of type B [19]. Large B-cell lymphomas with an anaplastic morphology are believed to represent a morphologic variant of Diffuse Large B-cell Lymphoma (DLBCB), which is the most common and one of most heterogeneous types of non-Hodgkin’s lymphoma [20,21]. The D430B cell line has been previously used in different studies to determine the activities of anti-CD30-immunotoxins in conventional 2D cultures [10,19,35]. In this study we aimed to determine the activity of BV in the CD30^+^ D430B cell line cultured in 3D, in a model that may therefore more closely recapitulate the structural features of a lympho-node with its microenvironment. In recent years, 3D model cultures have been the subject of a great deal of attention with respect to many types of cancer, mainly in solid tumors such as carcinomas of different histotypes [36,37,38]. Although the descriptions of the derivation of 3D model cultures of lymphoma are more limited, significant progress has recently been made in this field [39,40,41,42,43,44,45]. One of the major criticisms of generating 3D structures of lymphomas is that cells obtained from lymphoma patients, or either lymphoma cell lines, tend not to aggregate and do not spontaneously form compact and cohesive spheroids. For this reason, extracellular matrix such as collagen or even an optimized cytokine cocktail have been sometimes used to facilitate their aggregation [46,47]. Through bright-field inverted microscopy observations, we could instead appreciate that, in the case of the D430B cell line, the cells already appeared self-organized in spheroids, after 24 h of seeding in 96-well ULA plates. After another 24 h, the spheroids were quite homogeneous in their size and compactness (mean ± SD: 0.232 ± 0.005 mm^2^; *n* = 32), assuming a more defined round shape within 6–8 days. Furthermore, BV treatment was strongly effective in inhibiting spheroid enlargement and in inducing apoptosis, while Rituximab demonstrated very low activity.

Hybrid spheroids, generated by co-culturing HS5 stromal cells with D430B cells, in an attempt to recreate the lymph node microenvironmental niche, instead exhibited a less compact shape, where the stromal cells appeared aggregated in a round sphere surrounded by proliferating D430B cells, with a more disorganized arrangement. We further noted that a more compact arrangement was reached after 6–8 days of culturing. A gross evaluation of the hybrid spheroid area revealed an increase in dimensions, as compared with spheroids composed by D430B cells alone. A more detailed analysis further highlighted that the major contribution to the enhancement was effectively due to the D430B component (Supplementary Figure S2), thus suggesting that HS5 cells really support the proliferation of these lymphoma cells. Indeed, as already described in earlier studies, specific ECM features within the lymph node potentially acquired during neoplastic/MSC interactions [48], or primary stromal cells of the microenvironment [43], or either HS5 cells, as representative of the stromal component [49], sustain lymphoma cells’ survival. In agreement, we also demonstrated that the effect of BV on hybrid spheroids was significantly reduced. Apoptosis of D430B cells, as determined by Annexin/PI staining in the two types of spheroids treated with BV, confirmed in fact a protective effect by HS5 cells. Collectively these findings suggest that cells of the microenvironment may influence the drug response.

## 5. Conclusions

The choice of establishing 3D culture models of hemato-oncological malignancies, such as B-NHL, appears to be still of interest since they have not been widely developed to date. Although the 3D culture model of ALCL-B cells described here is rather simple, it might however represent a first step in the design of more complex models in the near future, potentially facilitating the development of personalized medicine for patients. Interestingly, this model could also be exploited to test new-generation ADCs, such as the recently developed anti-CD30 TUB-010 ADC, which shows higher stability and tolerability but lower cytotoxicity with respect to Adcetris, as proven in rodents and primate models [50]. Implementing the formation of simple spheroids with more complex 3D structures, also involving cells of the microenvironment (stromal and immune cells), thus appears pivotal in order to comprehensively determine the importance of the multiple interactions between neoplastic and surrounding cells taking place in vivo, potentially inducing resistance to therapy. Furthermore, combinations of multiple cells, such as the addition of T cells or NK cells or monocytes to the 3D culture model described herein, could also provide insights into lymph node-related immune-regulatory events [25], impacting the search for future immunotherapy strategies for B-Anaplastic Large Cell Lymphoma and other hematologic malignancies. The opportunity of performing gene expression profile studies comparing 3D in vitro cultures with in vivo-derived primary cells will enable further validation of these pre-clinical screening systems, as reported by Faria C. and co-authors regarding a 3D culture model of follicular lymphoma that they developed [41]. Finally, to the best of our knowledge, this is the first description of a 3D model of ALCL-B cells aimed at evaluating the activity of BV.

## Figures and Tables

**Table 1:** STR profile of D430B cell line.

STR Locus	D430B
D5S818	12,13
D13S317	11,12
D7S820	10,11
D16S539	12
VWA	18
TH01	6,10
AM	x,y
TPOX	9,11
CSF1PO	10,11
D21S11	30,32.2
D3S1358	15,17
D18S51	15,17
Penta E	7,12
Penta D	10
D8S1179	13,15
FGA	21,22

Fifteen highly polymorphic STR loci plus amelogenin (AM) were used.

## Data Availability

The data presented in this study are available on request from the corresponding author.

## Supplementary Materials

The following supporting information can be downloaded at https://www.mdpi.com/article/10.3390/antib14040098/s1, Figure S1: Different methods employed to generate D430B spheroids; Figure S2: Schematic representation of the method used to generate and to treat spheroids (D430B or HS5 only) and hybrid spheroids (D430B+HS5 cells); Figure S3 Phenotypic profile of HS5 cell line by cytofluorimetric analysis.
