# Supplementary material for: Developing a 3D Model Culture of an EBV+/CD30+ B-Anaplastic Large Cell Lymphoma Cell Line to Assay Brentuximab Vedotin Treatment
Source: Antibodies (Basel). 2025 Nov 10;14(4):98. doi: 10.3390/antib14040098 (PMC12641779; doi:10.3390/antib14040098)
Supplement: Supplementary file 1 [file antibodies-14-00098-s001.zip › antibodies-3841779-supplementary/Legends to supplementary Figures.pdf]

**Legends to supplementary Figures:**

**Supplementary Figure S1: Different methods employed to generate D430B spheroids: A.** Images relate to the 48hrs-culture of various number of D430B cells seeded in low attachment 96-U bottom plates with /without the addition of different concentrations of type 1 collagen. $5x10^3$:5K, $10x10^3$:10K, $20x10^3$:20K. **B.** Images relate to spheroids obtained by seeding $10x10^3$ (10K) D430B cells in a drop of 40µl of complete medium allocated in the lid of a culture Petri plate and mantained in the inverted position (hanging drop method) **C.** Images relate to the 48hrs culture of $5x10^3$ (5K) D430B cells/well seeded in ultra low attachment (ULA) 96 U-bottom-plate. For all images reference dimensional bar (white bar) is 60 µm.

**Supplementary Figure S2: Schematic representation of the method used to generate and to treat spheroids (D430B or HS5 only) and hybrid spheroids (D430B+HS5 cells).** $3x10^3$ D430B cells or $2x10^3$HS5 cells were seeded in each well of a ULA plate (spheroids). For generation of hybrid spheroids $2x10^3$ HS5 were cultured together with $3x10^3$ D430B. **B** Spheroids and hybrid spheroids obtained after 48hrs of culture in ULA plates were further treated with Brentuximab-Vedotin **(**10µg/ml**)** for 120hrs (48hrs +72hrs of treatment) when images were acquired. Red arrows and dashed line delineate the HS5 inner cell mass. White arrow and dashed white lines delineate area devoid of cells. Remainig green dashed lines identify the evaluated residual area of each spheroid/hybrid spheroid **D.** Histograms are representative of the values of areas of D430B or HS5 in spheroids or in hybrid spheroids calculated as reported above (**C**). Data are the mean ± SD of 4 samples for each spheroid type.

**Supplementary Figure S3: Phenotypic profile of HS5 cell line by cytofluorimetric analysis:** Histograms show the expression of CD73, CD105 and CD90 antigens in HS5 cells.
