# Supplementary figures and images for: Developing a 3D Model Culture of an EBV+/CD30+ B-Anaplastic Large Cell Lymphoma Cell Line to Assay Brentuximab Vedotin Treatment
Source: Antibodies (Basel). 2025 Nov 10;14(4):98. doi: 10.3390/antib14040098 (PMC12641779; doi:10.3390/antib14040098)

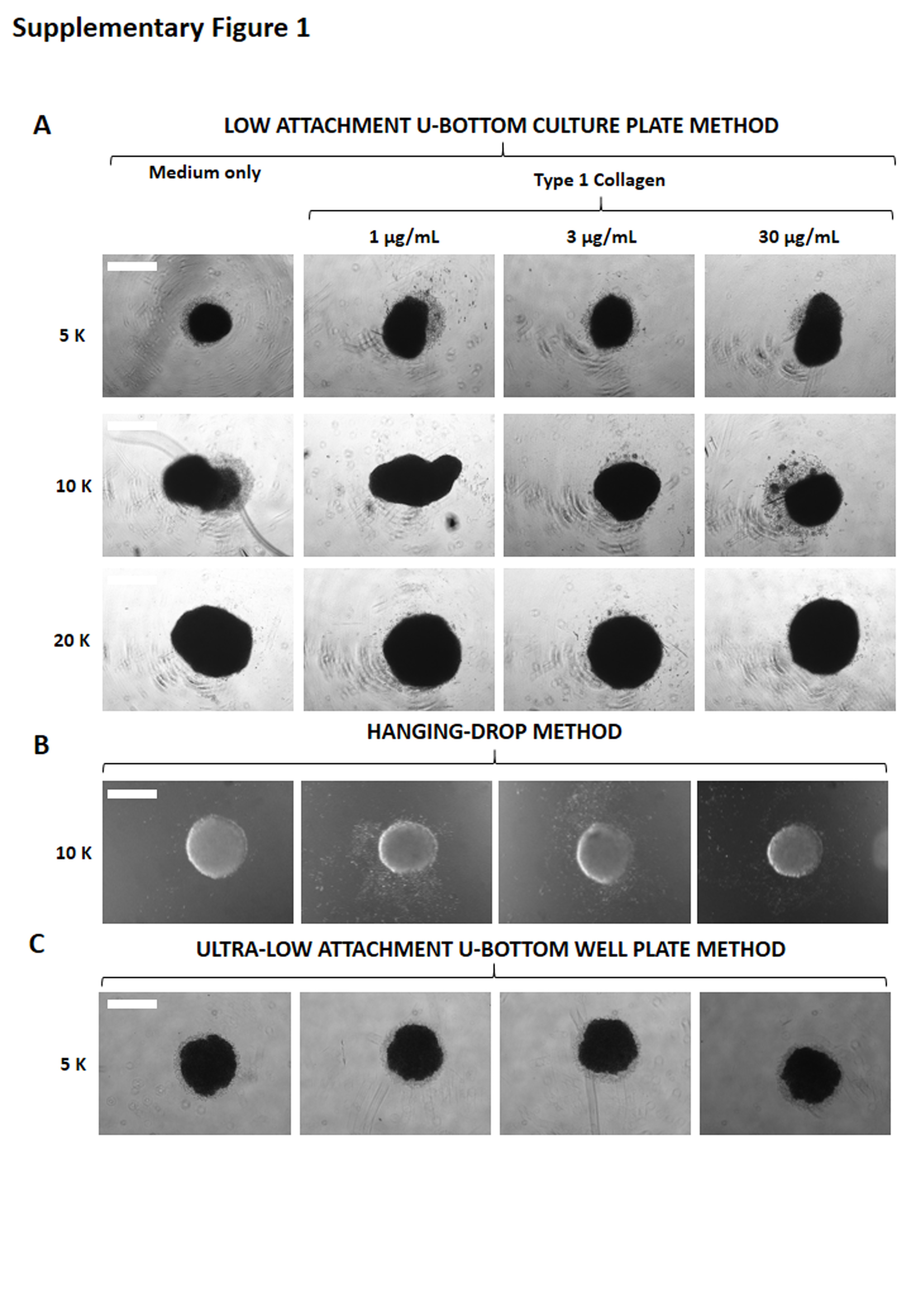

Supplement: Supplementary file 1 [file antibodies-14-00098-s001.zip › antibodies-3841779-supplementary/SupplementaryFigure1.png]

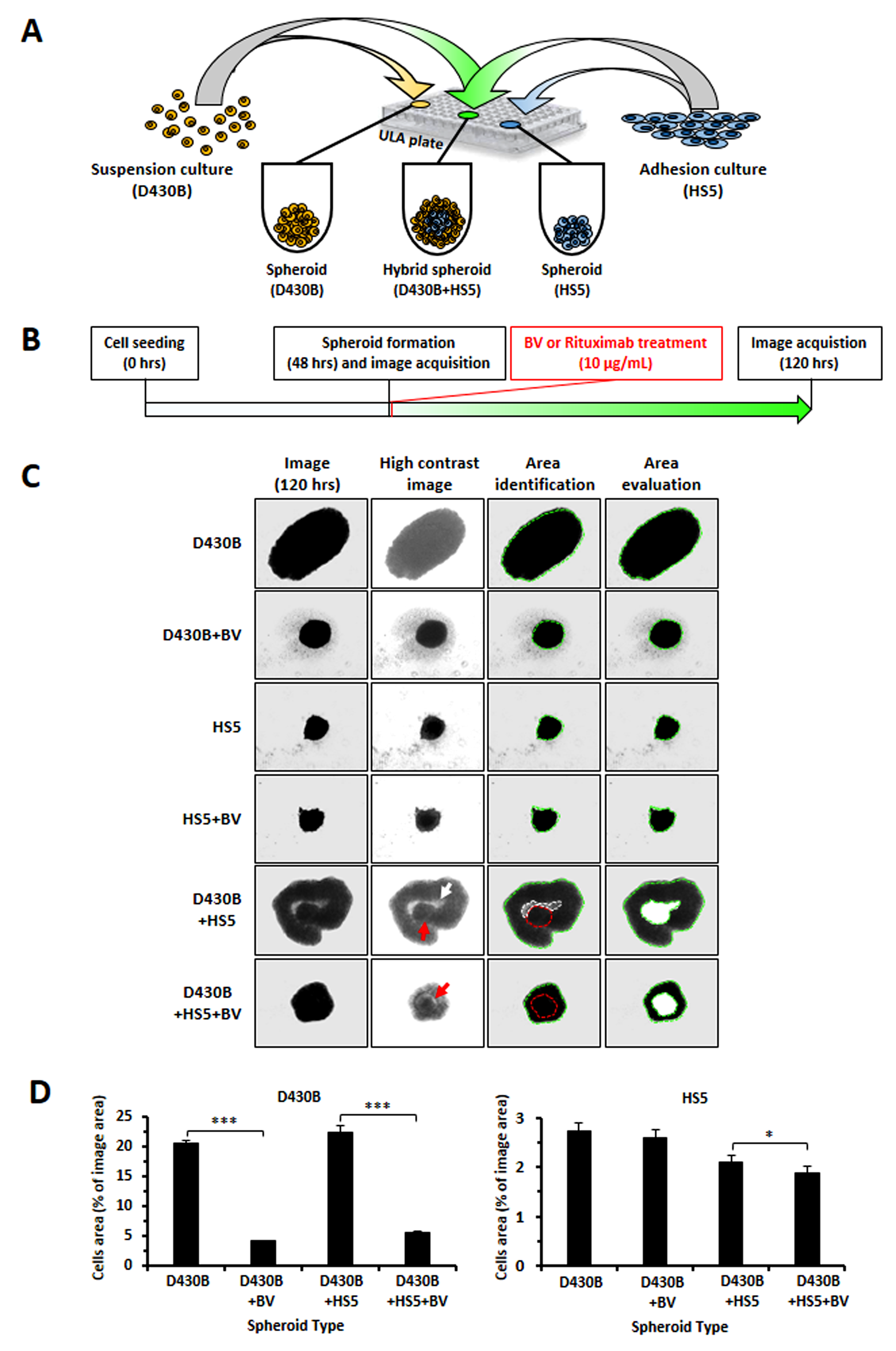

Supplement: Supplementary file 1 [file antibodies-14-00098-s001.zip › antibodies-3841779-supplementary/SupplementaryFigure2.png]

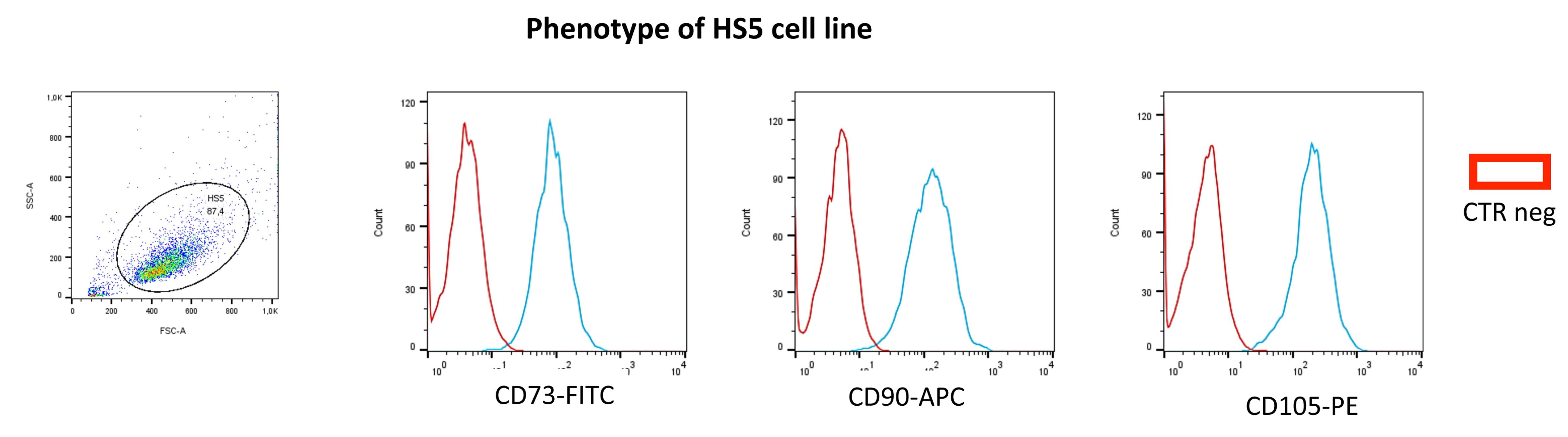

Supplement: Supplementary file 1 [file antibodies-14-00098-s001.zip › antibodies-3841779-supplementary/SupplementaryFigure3 .png]
